# Gender reassignment and neurosurgical implications: a narrative review

**DOI:** 10.1186/s41016-026-00447-5

**Published:** 2026-09-09

**Authors:** Rajdeep Brahmachari, Chandrasekaran Kaliaperumal

**Affiliations:** Department of Clinical Neuroscience, Royal Infirmary of Edinburgh, Little France, Edinburgh, UK

**Keywords:** Gender dysphoria, Gender-affirming hormone therapy, Transgender, Neurosurgery, Perioperative considerations, Transmen, Transwomen

## Abstract

Gender dysphoria is characterized by a marked incongruence between an individual’s experienced gender and assigned sex at birth. The global prevalence of transgender individuals is increasing, leading to greater interaction with healthcare systems across multiple specialties, including neurosurgery. Gender-affirming hormone therapy (GAHT), a cornerstone of treatment, has several neurological and neurosurgical implications. Nevertheless, neurosurgeons often report limited confidence in managing transgender patients because of insufficient awareness and training. This review explores the growing evidence of the broad neurobiological basis of gender identity, which involves variations in neuroanatomy and its contribution to the pathophysiology of gender incongruence. Moreover, the understanding of the neurovascular and neuropsychiatric effects of GAHT has provided a broad picture of the conditions under which a transgender patient can present to the neurosurgical outpatient department or emergency department. The review also discusses a holistic neurosurgical care plan for transgender patients (including gender dysphoria), which involves making a safe and conducive environment for the patient to open up during consultation, a proper method to obtain informed consent on the basis of the WPATH SOC 8 guidelines, and the identification of general and specific risk factors during the perioperative period and the subsequent optimization. Furthermore, this review identifies the factors that may affect proper intraoperative care such as anesthetic and antiviral drug interactions with GAHT hormonal agents, patient positioning and preparation issues, and thromboprophylaxis. This review provides an outlook for optimum postoperative care involving comfortable patient recovery for proper wound healing, pain management, and mental health support. In conclusion, transgender patients present unique challenges in neurosurgical practice owing to the combined effects of GAHT and psychosocial determinants. There is a critical need for improved education, inclusive clinical practices, and multidisciplinary collaboration to optimize patient outcomes. Further large-scale studies are needed to strengthen the evidence base and guide standardized neurosurgical care for this population.

## Background

Gender dysphoria is defined in the DSM-5 as marked incongruence between one’s experienced/expressed gender and their assigned gender, lasting at least 6 months. While gender dysphoria is a psychiatric diagnosis, the term “transgender” is used to refer to a person whose sex assigned at birth (usually based on the appearance of external genitalia) does not align with their gender identity (one’s psychological sense of their gender). It is estimated that there are 25 million transgender individuals worldwide [1]. Determination of the exact prevalence is difficult because of geographical differences and variations in the defining criteria, but some reports have suggested an increase in the prevalence of individuals seeking gender dysphoria care; for example, the prevalence of Americans identifying as transgender has doubled over the last 10 years [2], varying between 0.3 and 0.76% of the total population. Similarly, a retrospective study conducted in a Dutch gender identity clinic concluded that the number of people with gender identity issues seeking professional help has increased dramatically in recent decades [3]. This gradual increase has been consistent with the findings of epidemiological studies across various countries [4, 5]. The greatest increase in gender dysphoria has also been reported among children and adolescents [6].

Evaluation of the above epidemiological data indicates the need for a broader understanding of this condition and its primary treatment modalities. The increased prevalence also suggests that we are more likely to encounter gender dysphoria patients in a pre-, peri-, or post-gender-affirming treatment phase, and seek consultations in other relevant specialties, including neurosurgery. Several studies have indicated that patients with gender dysphoria who are undergoing gender-affirming hormone therapy (GAHT) are susceptible to attacks such as migraine, while the risk factors for secondary headaches are increased due to probable prolactinoma or meningioma [7]. The prevalence of epilepsy and the risk of cerebrovascular diseases have also been reported to increase [7]. Studies have concluded that neurosurgeons have expressed lower confidence in treating LGBTQ patients because of their limited knowledge and awareness of their condition [8]. Therefore, the aim of this review is to document and discuss studies that provide clarity about neuroanatomical variations, neurological and neurosurgical risk factors, and perioperative, neuroanesthetic, and neuropsychiatric considerations for evidence-based holistic neurosurgical care.

## Methods

A narrative review of the literature was conducted using peer-reviewed articles, retrospective studies, case reports, and other reviews indexed in databases such as PubMed, Google Scholar, EMBASE, and The NHS Scotland Knowledge Network. Information was also gathered from websites such as Psychiatry.org and WPATH. Search terms included important keywords like “Gender Dysphoria,” “Transgender Health,” “Gender Affirming Hormone Therapy,” “Neurological care in Transgenders,” and “Perioperative Care in Transgenders.” Some articles were accessed through OpenAthens account of the authors while other articles were available as open access. Finally, proof reading and editing were done by authors with the help of English language editing services available at Springer Nature provided by Curie.

## Transgenderism and its neurological relevance

There is growing evidence of a broad neurobiological basis of gender identity. Moreover, treatment modalities offering gender-affirming care have been found to have implications for the neurovascular health of these patients.

### Neuroanatomy

Neuroanatomical studies have shown that components of the brain of transgender individuals resemble the gender they identify with as opposed to the gender they are assigned at birth. Neuroimaging studies have also revealed increased cortical thickness and weaker connections in regions of the brain known for processing one’s own body perception [9, 10]. Brain connection dynamics are also reported to be altered, with similar dynamics in transgender individuals and the gender they identify rather than the gender they were assigned at birth [11]. Compared with both male and female controls, patients who are receiving gender-affirming hormone therapy (GAHT) present significant increases in gray matter density (GMD) and volume, specifically in the fusiform gyrus, cerebellum, and lingual gyrus [12]. While more studies need to be performed to further understand neuroanatomy and neurophysiology, the current research suggests that brain architecture and function play important roles in the pathophysiology of gender dysphoria and transgenderism.

### Effects of gender-affirming care and treatment modalities

The treatment for gender dysphoria is often multidisciplinary and combines the efforts of several behavioral health professionals and medical professionals. Two broad categories of treatment options are available: nonoperative and operative. Nonoperative treatment focuses on implementing psychosocial therapy and/or medical management with hormone replacement therapy. Operative treatments range from small cosmetic procedures to much larger genital transformation surgeries [13]. GAHT involves the administration of exogenous feminizing and masculinizing hormones such as estrogen, testosterone, and gonadotropin-releasing hormone (GnRH). Even patients who are planning to undergo gender-affirming surgery (GAS) are required to receive some form of hormone therapy as per the WPATH guidelines [2].

#### Headaches

One major study suggested that transgender people may experience more disabling headaches than cisgender people do (OR 1.76, 95% CI 1.34–2.32) [14]. An increase in the frequency of migraine has also been reported in patients receiving high-dose estrogens. This retrospective study also revealed that, among 73 transgender individuals, 23.4% of those who were transwomen-treated developed worsening of chronic pain, including headache, after at least 1 year of GAHT. Conversely, 23.1% of the transmen participants experienced improvement [15].

#### Benign brain tumors

A retrospective chart study revealed that gender-affirming hormone therapy (GAHT) is associated with a greater risk of meningioma and prolactinoma in transwomen, which may be linked to cyproterone acetate usage, and somatotropinomas in transmen. The study further reported that the incidence of meningioma was greater in transwomen than in the general European female population (standardized incidence ratio 4.1, 95% confidence interval 1.9–7.7) and the male population (11.9, 5.5–22.7). Like meningiomas, prolactinomas occurred more often in transwomen than in general Dutch females (4.3, 2.1–7.9) and males (26.5, 12.9–48.6). In transmen (female sex assigned at birth, male sex identity), two cases of somatotropinomas were observed, which was higher than expected on the basis of the reported incidence rate in a general European population (incidence rate females = incidence rate males; standardized incidence ratio 22.2, 3.7–73.4) [16].

#### Intracranial hypertension

An emerging and clinically significant observation relates to intracranial pressure dynamics in transgender individuals receiving hormone therapy. This case report and literature review identified 19 transmen patients with intracranial hypertension presenting with symptoms of headache, transient visual obscurations, and blurred vision. These symptoms were concurrent with the onset of exogenous testosterone therapy in 89.5% of patients [17], suggesting a notable correlation of clinical significance. There has also been a safety label warning issued by the US Food and Drug Administration in 2022 in the context of GnRH agonist use increasing the risk of idiopathic intracranial hypertension [7].

#### Cerebrovascular health

Studies have indicated that transgender individuals have an increased risk of cerebrovascular events such as stroke. A large cohort study of 3325 transwomen participants receiving GAHT revealed that the average incidence rates for ischemic stroke were higher among those with 6 or more years of hormone therapy (9.4 cases per 1000 person-years, 95% CI 5.2–17.0) than among those with less than 6 years (2.2, 95% CI 1.4–3.3) [18]. Several factors such as gender minority social stressors and the frequency of atypical mechanisms of stroke might contribute to increasing the risk of stroke with GAHT. Compared with cisgender people, those who identify as transgender may have increased tobacco and alcohol use, lower diet quality, and lower physical activity levels [19]; however, these data can be discrepant because of variance in sampling techniques and methodology between studies.

A meta-analysis of 14 studies performed in 2021, which included 109 transwomen participants, reported that ischemic stroke was the most common subtype of stroke in these patients, and the participants were on estradiol therapy [20].

#### Epilepsy

A retrospective cohort study of Medicare beneficiaries reported a higher prevalence of epilepsy in transgender individuals [21]. While no studies are available on the pathophysiology of epilepsy in transgenders, some reports suggest drug interactions between epileptic drugs and GAHT therapeutic agents [22].

## Neurosurgical care in transgender patients

### Safe and welcoming clinical environment

Neurosurgeons, as well as other professionals associated with delivering neurosurgical care, must ensure a safe and welcoming clinical environment. Studies have indicated that many individuals postpone their approach to medical care due to stigma, social isolation, and prejudice [23], and it has even been reported that some individuals are concerned with the doctor’s reaction to their gender identity [24].

### History taking and assessment

Neurosurgical care providers must ensure the use of inclusive language while communicating with a transgender patient. As inferred from the studies that we have evaluated, documenting the history of the type and duration of the GAHT treatment that the patient is currently undergoing is crucial. Several neurological effects of GAHT hormone therapy may mimic symptoms of neurosurgical pathologies or even aggravate symptoms. It is important to understand the ideas, concerns, and expectations of patients regarding the reason they are presenting or referred for neurosurgical consultations. Once an open connection is established with the patients and they are assured that they are in a safe and comfortable space, neurosurgeons should provide treatment that addresses their unique health challenges [25].

### Medicolegal aspects and consent

Informed consent for neurosurgical procedures must be obtained after the mental health status of the concerned patient is evaluated and considered. A study states that before informed consent is obtained, it is necessary to evaluate the biological, social, and psychological risks. It also asks us to reflect on 2 important questions: “Does the patient have a clear idea of the risks of the services that are being requested? Is the consent truly informed?” [26]. The WPATH SOC 8 recommends patient autonomy in making decisions but suggests the evaluation of cognitive capability, addressing cooccurring mental health conditions and mental health professional advice before providing informed consent [27].

### Preoperative optimization

It is highly important to verify accurate patient particulars by documenting patients’ preferred names, preferred gender identities, pronouns, and terminology [28, 29].

It has been reported that at least 80% of transgender patients are currently receiving or are interested in receiving gender-affirming hormone therapy, which must be evaluated because some of these regimens are associated with surgical risks [30], including neurosurgery (as highlighted in the Effects of gender-affirming care and treatment modalities section). The regimens of GAHT agents must not be stopped, as they can put patients at psychological and physical risk [31].

With respect to general perioperative risks, studies have shown that transmen and transwomen are at increased risk of HIV, human papillomavirus, hepatitis B, and methicillin-resistant *Staphylococcus aureus* infections [32, 33]. Moreover, compared with the general population, transgender individuals are more likely to experience distress, anxiety, and depression, which might predispose them to smoking, alcohol consumption, and drug abuse, leading to increased perioperative risk [33]. Studies have also suggested a predisposition to diabetes and insulin resistance in transgender patients [21], but further studies are warranted (Table 1).

**Table 1:** Summary of the specific biochemical changes and possible neurological problems that arise from the effect of GAHT therapy, which contributes to preoperative risks (along with literature references)

Patients	GAHT agents	Coagulation parameters	Hematological changes	Biochemical changes	Neurological relevance
Transgender men (people who were assigned female at birth but identifies and lives as a man)	Dissolved testosterone crystals (gel and patch); testosterone cypionate; testosterone enanthate; testosterone undecanoate [34]	Increase in factor IX and protein S, increase in hematocrit [35] Increased risk of venous thrombosis [34]	Increased hemoglobin, erythrocytes, hematocrit [35]	Increased creatinine, increased cholesterol and triglyceride, increased transaminases [35]	Intracranial hypertension [17], tumors: prolactinoma, somatomammotropinoma [16]
Transgender women (people who were assigned male at birth but identifies and lives as a woman)	Ethinyl estradiol; 17-β-estradiol; medroxyprogesterone; transdermal patch with norethindrone; cyproterone; spironolactone [34]	Increased factor II, VII, VIII, X, fibrinogen [35] Increased risk of venous thrombosis [34]	Decrease in hemoglobin, erythrocytes, and hematocrit [35]	Increased cholesterol and triglyceride, decreased calcium, increased risk of hyperprolactinemia [35]	Migraine [15], cerebrovascular stroke [20] Tumors: meningioma, prolactinoma [16] Glioblastoma [36]

Specific considerations must be given to pregnancy tests, thromboprophylaxis for venous thromboembolism [34], and the optimization of any comorbidities through multidisciplinary approaches involving endocrinology, neuropsychiatry, anesthesia, neurology, and gynecology inputs.

### Intraoperative considerations

In this section, it is important to highlight the implications of anesthesia along with other intraoperative considerations. Patients taking feminizing hormone therapy may encounter interactions when taking antiseizure medications, corticosteroids, and neuromuscular blockers, such as succinylcholine. Perioperative neuromuscular monitoring must be performed when the neurosurgical procedure requires the use of neuromuscular blocking agents [37]. HIV-positive patients who receive antiviral treatment may experience drug interactions with certain hypnotics, anxiolytics, and antibiotics [34].

Intraoperative blood transfusion must be considered with caution because of the hematological changes due to the effect of GAHT; however, there are no significant studies regarding the implications of GAHT for blood transfusion in transgender patients.

During airway management, it is important to allow for chest expansion during surgery; for example, a study inferred that transmen who have not undergone gender-affirming surgery may use breast binders or other garments that could restrict respiration, causing severe respiratory complications [34]. For transwomen, orotracheal intubation can be a matter of concern if they have undergone procedures involving the larynx such as laryngoplasty [34].

While considering intraoperative urinary catheterization, some transgender patients who have undergone gender-affirming surgery (GAS) might require a vesical catheter, and consultation with urology or another specialized service that is well-versed in the urogenital anatomy in relation to that surgery is recommended [34].

Healthcare providers must be vigilant and follow standard precautions to prevent the transmission of HIV for the safety of patients and healthcare, when caring for an HIV-positive patient [34].

### Postoperative considerations

Postoperative care must consider several parameters, including wound healing, pain management, and potential complications.

After satisfactory recovery, room assignments must be prioritized; if a private room is available, it should be offered as an option because increased privacy may provide additional comfort and/or safety for the patient. However, offering a private room as the only option should be avoided because it may cause patients to feel isolated instead [38].

When caring for transgender neurosurgical patients, it is essential that the healthcare staff attending to them during their recovery consistently acknowledge their preferences regarding their name and pronouns; this is crucial for avoiding psychosocial stressors that can delay their recovery and reintegration into their regular environment. A better patient–physician connection has been linked to better adherence to medical advice following hospitalization [39].

Wound healing must be assessed and prioritized. In transgender patients, the process of scar formation is heavily influenced by the patient’s risk factors. Keloid and hypertrophic scars can have significant psychosocial implications; hence, these scars require appropriate wound care [40]. The recovery of neuromuscular and bone health after spinal surgery might not be difficult, as studies have shown improvements in bone health with both masculinizing and feminizing GAHT [41]. In contrast, in the case of the gonadotropin-releasing hormone agonist regimen, the participation of an endocrinologist is essential for monitoring bone mineral density and providing recommendations to reduce the risk of vertebral fractures following spinal surgery through calcium and vitamin D supplementation [42].

Postoperative pain management must be performed adequately through a multidisciplinary approach and appropriate analgesics to improve patient outcomes. Pain control techniques can include pharmacological, regional anesthetic, and physiotherapy [43]. The employment of gender-affirming hormone therapy (GAHT) has a neuroprotective effect on spinal cord injuries, especially estrogens, which can reduce neuropathic pain [44]. However, there are few publications on the comprehensive pain management of transgender patients.

Certain measures must be considered, such as administering antiemetic prophylaxis and implementing postoperative prophylaxis for deep venous thrombosis (Table 2). Notably, the use of spironolactone may lead to orthostatic hypotension [45].

**Table 2:** Summary of the postoperative considerations mentioned above (BMD = bone marrow density, low-molecular-weight heparin reference mentioned above)

	Comfortable recovery	Postoperative pain management	VTE prophylaxis	Bone health (in case of spinal surgeries)	Wound healing	Mental health
Postoperative considerations	Room assignment preferably private room in the hospital Health staff acknowledge their preferences regarding their name and pronouns; crucial for avoiding psychosocial stressors	Multimodal and multidisciplinary approach Pharmacological, anesthetic, and physiotherapy options GAHT has neuroprotective effect and reduces neuropathic pain	To be considered during preoperative phase and continued during postoperative phase Low-molecular-weight heparin can be considered along with other anti-coagulants [34]	GAHT can assist bone health recovery In case of GnRH agonists, assessment of BMD, involvement of endocrinologist and calcium and vitamin D supplementation	Appropriate wound care, especially keloid and hypertrophic scars Aseptic dressing and antibiotics	Avoid prolonged in patient stay and aim for early mobilization prevent worsening of anxiety and depression Neuropsychiatry follow-up and advice

As mentioned earlier, transgender patients are predisposed to anxiety and depression [46], which can worsen and impact mental health post-surgery due to prolong inpatient hospital stays [47].

## Conclusion

This review suggests that there is a wider need for education and awareness about transgender health and its implications for various medical and surgical specialties, including neurosurgery. The centers of excellence in neurosurgery must understand the importance of education about transgender health and become proactive in imparting training to healthcare providers involved in neurosurgical care delivery. This review also concludes that transgender patients undergoing GAHT may present with specific symptoms relevant to neurology and neurosurgery, which may arise from psychobiological and psychosocial factors. Moreover, neurosurgeons must be aware of their transgender patients, execute proper history-taking and examination procedures, and identify available stakeholders for consultation to obtain proper informed consent. Neurosurgical care providers should also be informed about perioperative changes and their associated risks. Despite the availability of extensive studies in healthcare, evidence and data related to transgender-related needs have been mostly limited to short retrospective studies, case reports, and reviews. Research and studies regarding transgender health must continue to be published for a broader understanding of transgender healthcare.

## Data Availability

No datasets were generated or analysed during the current study.

## Abbreviations

GAHT: Gender-affirming hormone therapy
WPATH: World Professional Association for Transgender Health
GAS: Gender-affirming surgery
BMD: Bone mineral density
TGD: Transgender
GMD: Grey matter density
GnRH: Gonadotropin-releasing hormone
DSM: Diagnostic and Statistical Manual for Mental Disorders
